# Ogilvie's Syndrome in a Male With Bladder Cancer

**DOI:** 10.7759/cureus.55097

**Published:** 2024-02-27

**Authors:** Jacqueline T Wesolow

**Affiliations:** 1 Internal Medicine, Moffitt Cancer Center, Tampa, USA

**Keywords:** colon distention, massive bowel dilation, bladder cancer, bowel distention, ogilvie's syndrome

## Abstract

Ogilvie's syndrome is characterized by massive distention of the colon without evidence of physical obstruction. This condition can present itself with abdominal distention that is difficult to treat and can lead to ischemia of the bowels and, ultimately, bowel perforation. Treatment is aimed at supportive care with decompression, intravenous fluid hydration, and symptom control. This is a case presentation of Ogilvie's syndrome in an 80-year-old male with bladder cancer. He presented with abdominal distention and was found to have massive dilatation of his colon without evidence of obstruction on a CT scan. He was managed with supportive care, including stool softeners, and ultimately placed on total parenteral nutrition.

## Introduction

Ogilvie’s is a condition characterized by massive colonic distention that is not due to a mechanical obstruction [1]. These patients do not have any mass or physical blockage causing the distention; hence, it also being called a pseudo-obstructive process. Diagnosis is based on radiographic findings showing colonic distention [1,2]. The distention, if severe enough, can ultimately lead to ischemia or colon perforation, and when that occurs, it has a high mortality rate of ~ 40% [2]. Given the high morbidity and mortality associated with this condition, it is imperative to identify Ogilvie’s in patients. This is seen in the ill, immunosuppressed, or hospitalized patient population. Patients can present with abdominal distention, discomfort, nausea, or vomiting, among other symptoms. Treatment is aimed at supportive care, which includes intravenous fluid hydration, decompression of the abdomen and pain control [2,3].

## Case presentation

An 80-year-old male with a past medical history of stroke, atrial fibrillation, COPD, diabetes type 2, hypertension, obesity, obstructive sleep apnea, osteoporosis, and bladder cancer status post radical cystoprostatectomy with ileal conduit creation with ureteral stent that presented to our cancer center's urgent care with hematuria and left flank pain. The patient was recently admitted to our cancer center for two weeks with renal failure secondary to obstruction and displacement of his left ureteral stent. Stent was exchanged at that time. On this presentation, his left ureteral stent was found to be again displaced and coiled within the left ureter. He was subsequently admitted to our internal and hospital medicine services and successfully underwent a stent exchange with our interventional radiology team. Most of his electrolytes were close to or within the normal range (Table 1). 

**Table 1:** Initial laboratory values

Lab	Patient’s Value	Normal Range
Sodium	135 mEq/L	135 to 145 mEq/L
Potassium	5.5 mmol/L	3.6 to 5.2 mmol/L
Chloride	107 mEq/L	96 to 106 mEq/L
CO2	15 mEq/L	23 to 29 mEq/L
Creatinine	3.5 mg/dL	0.6 to 1.1 mg/dL
Phosphorus	4.1 mg/dL	2.8 to 4.5 mg/dL

**Figure 1:**
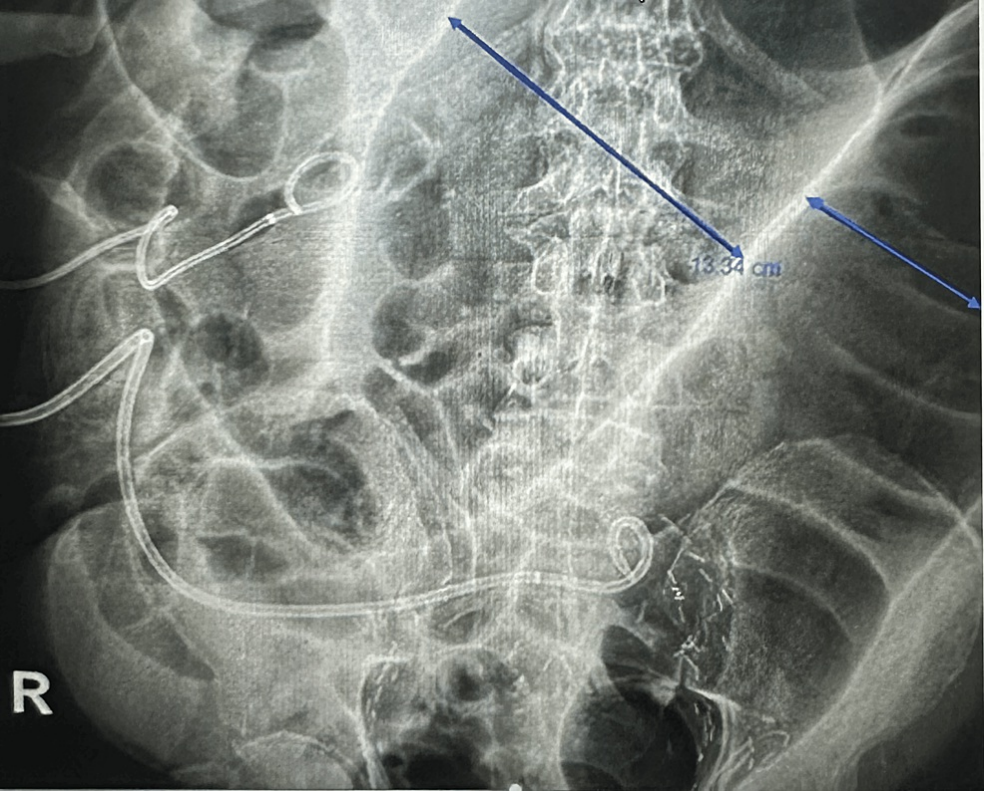
Abdominal X-ray showing extensive colonic distention up to 13.34 cm.

**Figure 2:**
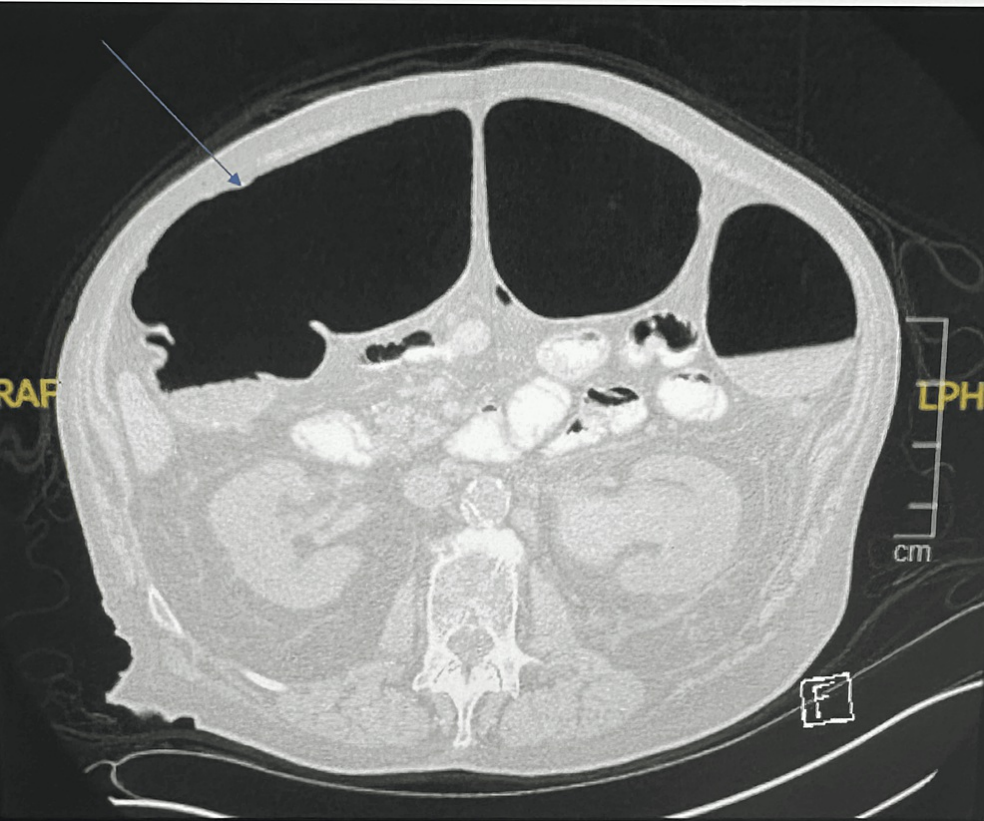
CT imaging of the abdomen and pelvis shows diffuse dilatation of the proximal colon.

His abdomen was noted to be severely distended. An abdominal X-ray showed extensive colonic distention measuring 11 cm in the descending colon and 13.34 cm in the transverse colon, with extensive postsurgical changes in the pelvis (Figure 1). The patient had persistent bowel dilation throughout his hospital course, with ongoing abdominal distention and significant bowel dilation. A CT scan of the abdomen and pelvis revealed diffuse dilatation of the proximal colon (Figure 2). No evidence of obstruction was noted on the CT scan, supporting the diagnosis of Ogilvie's syndrome. 

**Figure 3:**
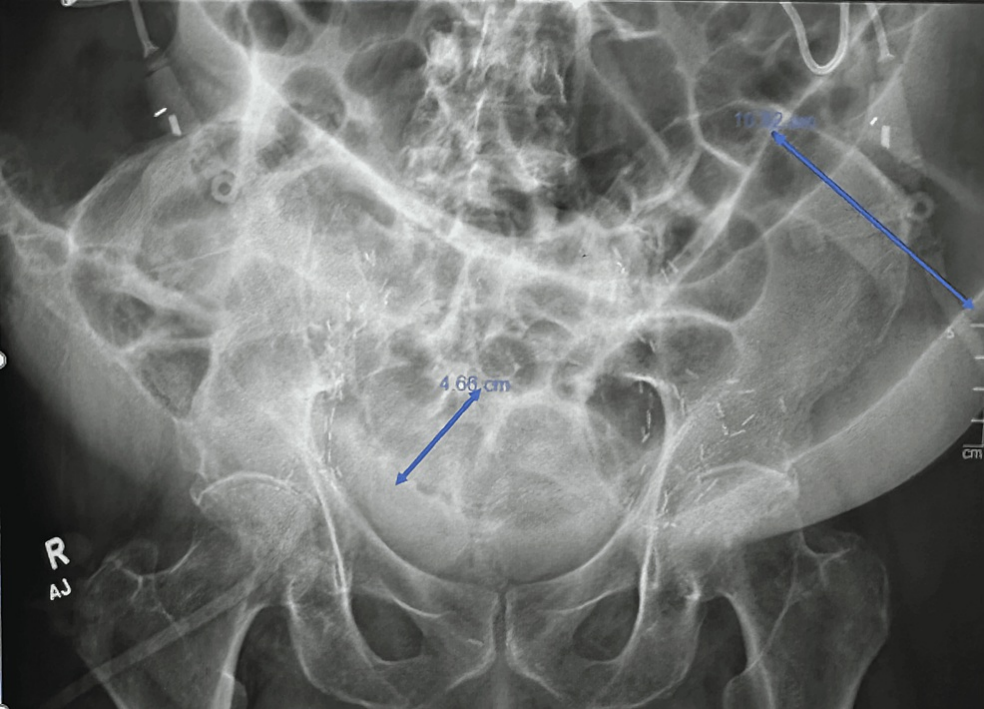
Abdominal X-ray showing severe colonic distention

**Figure 4:**
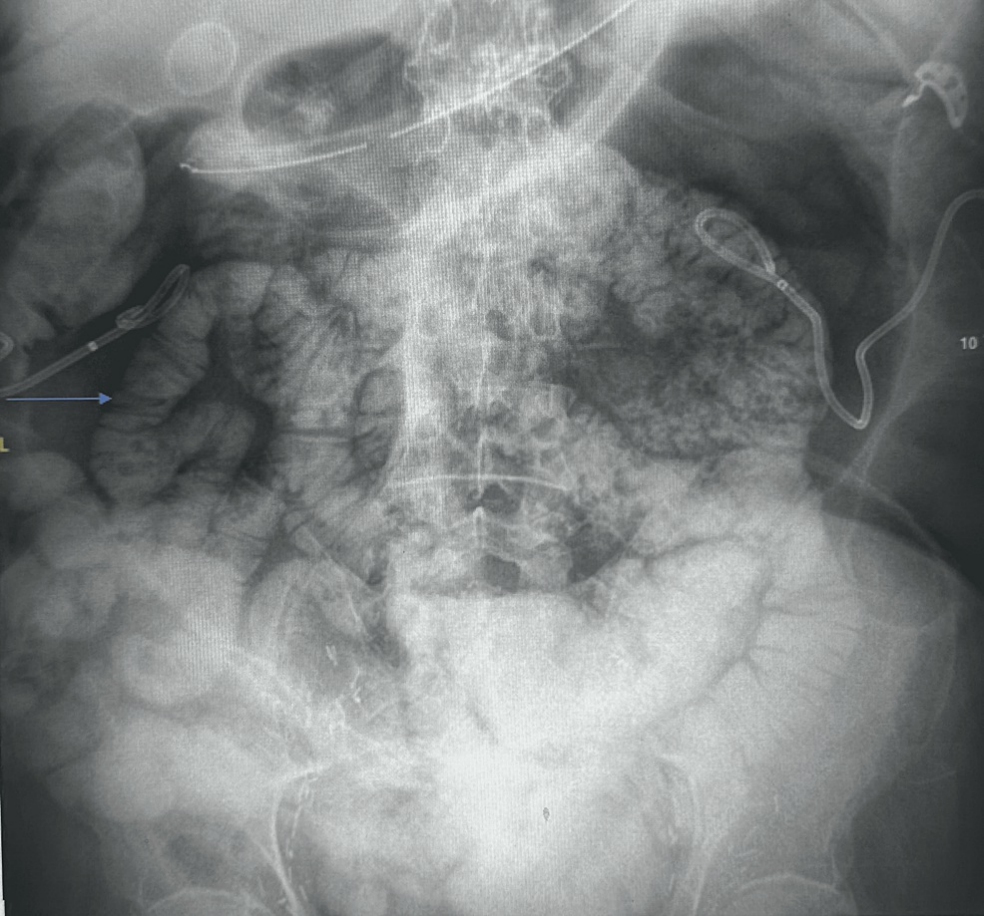
The small bowel follow-through study revealed contrast throughout without evidence of obstruction.

Repeated serial abdominal X-rays noted persistent bowel distention. An X-ray about two weeks into his hospital stay showed a small bowel loop in the pelvis measuring 4.7 cm, with the distal descending colon measuring 10.5 cm (Figure 3). The patient was seen by our gastroenterology surgery team for what was initially thought to be colonic ileus, likely multifactorial related to recent procedures, surgical history, and decreased activity. The patient had bowel movements. The surgery team at the time recommended that the patient remain nothing per oral with sips of clears. The patient had multiple follow-up scans for monitoring, which were also concerning for a possible ileus. He had a nasogastric tube inserted, which was eventually removed without improvement in bowel distention. He was started on total parental nutrition since a gastric tube placement was likely not to be successful, given the degree of bowel dilation and the lack of an adequate window for placement. Additionally, the patient underwent an upper gastrointestinal small bowel follow-through study that showed a normal stomach-to-cecum transit time of approximately two hours (Figure 4). No obvious gastric retention was seen in this study, further supporting the diagnosis of Ogilvie's syndrome. 

The patient was continued on stool softeners and miralax throughout the hospital stay. He remained dependent on total parental nutrition as his primary source of hydration and energy and was discharged from it. A detailed discussion was conducted with the patient and his family on the importance of following up with his primary care physician. 

## Discussion

Ogilvie’s syndrome carries a high morbidity and mortality risk and is therefore, an important diagnosis to identify early on. Can often be mistaken for ileus if not properly followed up with. This can lead to further distention of the abdominal walls and cause acute ischemia of the bowel, which can lead to a life-threatening abdominal perforation. Diameters of more than 14cm are associated with a higher risk of perforation [4]. Treatment consists of conservative management, including nothing per oral (NPO), intravenous fluid hydration, and symptom control. Due to the dehydration and malnutrition associated with this condition, electrolyte imbalances are often seen. Patients can present with, most notably, hypokalemia, hypocalcemia, and hypomagnesemia [5].

Monitoring can include follow-up serial abdominal series X-rays while the patient is in the hospital. In patients with a cecal diameter of more than 12cm or who have failed supportive management, neostigmine can be considered as a treatment option [6,7]. Decompression with a nasogastric tube is an option and can be helpful for patients who are severely distended with pain. If there is evidence of perforation, then endoscopic decompression and/or a surgical consultation can be considered. Colonoscopic decompression has been shown to decrease the cecal diameter in about 73%-100% of cases [1].

It is thought that neural processes lead to massive colonic distention, causing dysmotility [6]. Ogilvie’s has been reported after surgeries including orthopedic, obstetric, and urologic [6-7]. Pain medications such as opioids should be avoided as they slow down the transit of stool in the bowels, thus increasing the risk of fecal impaction and potentially leading to increased colonic distention. Also, drugs that decrease the motility of the gut, such as antidepressants, antihistamines, and antipsychotics, should also be minimized or avoided. These medications are part of the anticholinergic family of drugs that inhibit nerve impulses responsible for involuntary muscle action, thus decreasing colonic motility [7,8]. It is imperative to review all of the patient’s medications thoroughly.

Unfortunately, our patient could not tolerate much more than sips of clear liquids. Therefore, he required total parenteral nutrition as the main form of nutrition. Our patient stabilized while on total parenteral nutrition. This is a difficult disease process for patients and their families to live with. Consulting with a social worker or patient support team can assist with this disorder. Proper follow-up after patient discharge from a hospital setting with a primary care physician is key to ensuring optimal management.

Our patient case represents a classic clinical scenario of Ogilvie's syndrome. A literature review of another patient case describes a 71-year-old woman with Ogilvie's syndrome who presented with similar symptoms of abdominal distention and pain [3]. Her CT abdomen and abdominal X-rays revealed a parallel pattern of marked non-obstructive distention [3]. Of note, both cases represent a geriatric population.

## Conclusions

Ogilvie's syndrome is a rare disease process in which the colon becomes massively distended. It is thought that neural processes lead to the marked distention. Patients can present with abdominal distention, discomfort, and a wide range of electrolyte abnormalities, such as hypokalemia and hypomagnesemia. It is seen commonly in the elderly and hospitalized patient populations. It can lead to bowel ischemia and perforation; therefore, it is crucial to identify this as early on as possible. Just as in our case, treatment includes bowel rest, decompression, and supportive care. Continued close monitoring and follow-up is key to decreasing the risk of perforation and ensuring the best possible outcome for these patients. 
